# Reimbursement status, access delays and prices of orphan drugs in France: a population-stratified analysis of indications approved between 2021 and 2024

**DOI:** 10.1186/s13023-026-04533-w

**Published:** 2026-08-05

**Authors:** Izzat Sultan, Pascal Paubel, Albane Degrassat-Théas

**Affiliations:** 1 School of Pharmacy, Université Paris Cité, 4 avenue de l’Observatoire, Paris, 75006 France; 2 General Agency of Equipment and Health Products (AGEPS), Assistance Publique-Hôpitaux de Paris (AP-HP), 7 rue du Fer à Moulin, Paris, 75005 France; 3 Health Law Institute, INSERM, UMR S 1145, Université Paris Cité, 45 rue des Saints-Pères, Paris, 75006 France

**Keywords:** Access to drugs, Adults and children, Rare diseases, Reimbursement & pricing

## Abstract

**Background:**

More and more treatments are available for patients with rare diseases. However, a marketing authorization (MA) for a new treatment does not mean access in France for all patients. Moreover, while rare diseases affect children mostly, paradoxically it seems that they are the ones who faces more difficulties to access treatments. In this context, the aim of this study is to analyze the French market access of drugs in rare diseases recently approved between July 2021 and July 2024, depending on their target population (adults, children or both).

**Results:**

Overall, 43 orphan drugs were selected and 51 MA/extension of MA were analyzed. In the selected sample, there are four times fewer orphan drugs indicated only for children (7/51) than for adults (29/51). On 1 April 2025, 53% of these indications (27/51) were reimbursed in France with 57% being pediatric indications (4/7) and 48% being adult indications (14/29). Furthermore, the median delay between the granting of the MA and reimbursement through the standard procedure in France was 505 days. Even though there are longer delays of reimbursement and lower prices compared to the other four European largest pharmaceutical markets, France is the second country with the most indications reimbursed on 1 April 2025 behind Germany (80%, 41/51).

**Conclusion:**

On 1 April 2025, patients in France had partial access to orphan drugs recently approved, regardless of the target population. The prices and delays associated with the standard reimbursement procedure are factors that have a considerable impact on access to the French market for drugs intended for the treatment of rare diseases.

**Supplementary Information:**

The online version contains supplementary material available at https://doi.org/10.1186/s13023-026-04533-w.

## Introduction

The significant diagnostic and therapeutic progress seen these last years, has revived hope for patients living with rare diseases [1]. Indeed, due to their rarity, usually no treatment exists for these diseases, referred as orphan diseases. Rare diseases affect approximately 3 million individuals in France, with pediatric patients (i.e. children from 0 to 18 years old) representing the majority of cases [2]. Paradoxically, pediatric patients are the ones who faces more difficulties to access treatments due to the diagnosis of these diseases and to the complexities in drug development for children [3]. Moreover, whether it is adults or children, in case of a marketing authorization for a new treatment, access delay to drug reimbursement is often lengthy.

As treatments of rare diseases are expensive, access for patients is only possible if drugs are reimbursed by the national health insurance. In France, drugs reimbursement remains an important item of total expenditure. In 2023, drugs reimbursement cost 36.5 billion euros to the national health insurance (representing an expenditure increase of 9.3% compared to 2022). In addition, expenditure related to orphan drug reimbursement increased 28.3% compared to 2022 due to the strong sales of cancer immunotherapies such as Darzalex^®^, Yescarta^®^, Kymriah^®^, Abecma^®^ and Tecartus^®^. All distribution channels combined (hospitals and community pharmacies), reimbursement for orphan drugs represented 5 billion euros of total expenditure in 2023 [4]. Due to the economic and budgetary context in France, setting the prices of drugs for rare diseases is a major issue in the French market access process.

The aim of this study is to analyze the French market access of drugs for rare diseases approved recently, depending on their target population (adults, children or both). Most recent publications are focused either on the price of orphan drugs [5–11], or on the impact of the European regulation (EC) No. 141/2000 on the market access of drugs in rare disease [12–15] or on the consequences of the French derogatory access system reform in July 2021 for these medicines [16, 17]. However, these publications do not take a patient-population based approach which consists in stratifying results regarding the target population of the drug (adults, children or both).

This work based on a patient-population approach will enable to identify key barriers limiting access to reimbursement for these medications. In addition, a quick overview of access level to these drugs across the five largest European pharmaceutical markets (including France, Germany, Italy, Spain and United Kingdom) is presented. With all these elements, we will outline potential solutions to improve future access to treatments for rare diseases in both adults and children in France.

### Overview of market access procedure in France

In France, there are two types of market access procedures that allow the reimbursement of a drug. The first type is the standard reimbursement procedure. It concerns drugs with a marketing authorization delivered either by the European Commission (EC) or the French national agency for medicines and health products safety (Agence nationale de sécurité du medicament et des produits de santé, ANSM). For this procedure, access for patients is possible, only after a health technology assessment (HTA) of the drug, followed by a pricing and reimbursement rate setting of the medicine. It should be noted that HTA for all new drugs are carried out by the transparency committee (Commission de la transparence, TC) of the French Health Authority (Haute autorité de santé, HAS). French HTA includes an assessment of the drug clinical benefit (*Service médical rendu*,* SMR*) and its clinical added value (*Amélioration du service médical rendu*,* ASMR*) in a therapeutic indication. This last criterion is the basis for pricing negotiation and five levels of ASMR can be attributed depending on the therapeutic progress offered by the drug in a particular indication^1^. On the other hand, the Economic committee for Health Products (Comité économique des produits de santé, CEPS) is responsible for drugs pricing negotiations with pharmaceutical companies. Furthermore, the reimbursement rate for new drugs is set by the national union of health insurance funds (Union nationale des caisses d’assurance maladie, UNCAM) [18, 19]. This procedure ends with the publication in the Official Journal of the French Republic of the reimbursement and pricing decision made by the Minister of Health.

**Fig. 1:**
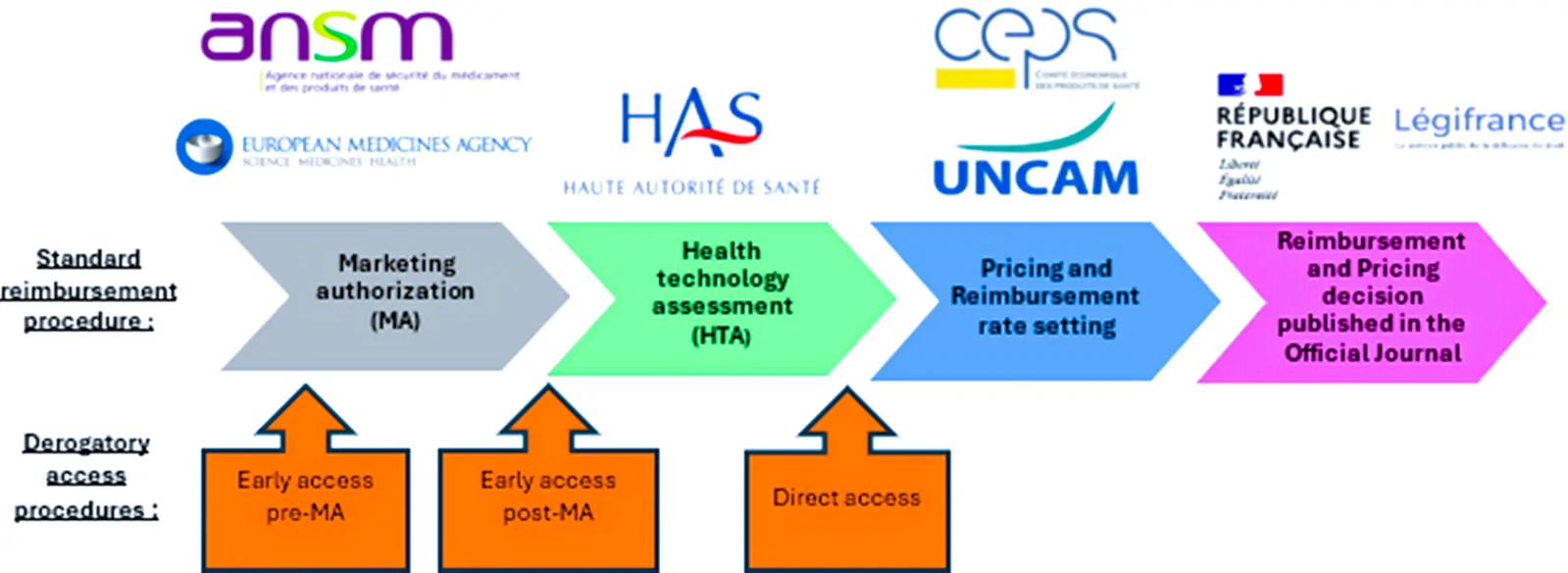
Main reimbursement procedures allowing market access for a drug in France

The second type is derogatory access (or early access) procedures which allow the temporary reimbursement of a drug based on promising clinical data. These schemes have been set up to accelerate early access for innovative medicines, especially for patients affected by serious and rare diseases. Following the reform of the derogatory access schemes in July 2021, two main types of early access are possible in France in addition of the compassionate use program: pre-MA early access and post-MA early access while HTA and pricing negotiation under standard procedures are still ongoing [20]. In addition to these schemes, in 2022, a “direct access” pathway experimentation was created highlighting the expansion of this type of program in response to difficulties in accessing the market [21]. This experimental procedure allows temporary reimbursement after a positive HTA during the price negotiation, which cannot exceed 10 months (Fig. 1).

Please note that these two types of market access procedures are the same regardless of the drug target population. However, the TC does consider specificities of drug development for pediatric population in its HTA process.

### French market access of drugs for rare diseases

So far, only few works focused on the French market access of drugs for rare diseases [22], although there are studies and reports on European market access of these medicines [23–26]. In 2010, ten years after the introduction of the European regulation (EC) No.141/2000 on orphan medicinal products, an inventory of access and prices of orphan drugs across Europe, was done for 60 orphan drugs approved in 10 member states [26]. This study showed that in France, 90% of these medicines were accessible but this proportion was reduced to 33% in some of the countries included in the study [26]. In 2019, a study focusing on the French market access of orphan drugs approved in Europe before August 2016, showed that 78.3% of therapeutic indications were reimbursed by the national health insurance and the median delay to French market access was 360 days [22].

## Materials and methods

This cross-sectional descriptive study focuses on orphan drugs approved between the 1 July 2021 and the 1 July 2024, indicated for adults and/or children affected by a rare disease [27]. We started to include orphan drugs approved on 1 July 2021, because it coincides with the implementation in France of the reform on drug derogatory access programs [20]. Drugs with no longer valid MA were excluded from this analysis. Also, medicines indicated for rare infectious diseases were also excluded as these diseases are described by “incidence” as the epidemiological indicator [28]).

French market access of these drugs was analyzed by indication using three access criteria (Table 1). Moreover, it should be noted that for our study “national public prices” was our third access criterion (Table 1). Even though it is not a direct measure of access, we believe that national prices must be examined as they affect orphan drug access to patients.

A secondary analysis, comparing access to these drugs between France and the four other largest European pharmaceutical markets (including Germany, Italy, Spain and United Kingdom) was performed using access criteria n°1 and n°3 (Table 1).

All analyses were descriptive and no inferential statistics were applied on the selected sample.

**Table 1:** Main access criteria used for the analysis of the French market access

	Access criteria Assessed	Details on the criteria	Scope of analysis
**Criterion n°1**	**Reimbursement** **on 1 April 2025**	A drug indication was considered reimbursed on 1 April 2025, if a positive decision was published for its standard reimbursement or for any derogatory access program. *NB*: In France, after the standard reimbursement procedure, drugs can be listed on several lists: - “*Assurés sociaux*” (for drugs delivered by community pharmacy) and/or, - « Collectivités *et divers services publics* » (for drugs delivered by hospital pharmacy) and/or, - “En *sus*”* (for some eligible drugs delivered in hospital pharmacy).	All orphan indications selected for analysis
**Criterion n°2**	**Access delay to reimbursement** **in France**	Three delays were studied: - Total delay of the reimbursement procedure (corresponding to the delay between the granting of a MA and the publication of the reimbursement decision); - HTA phase (corresponding to the delay between the granting of a MA and the publication of the TC opinion); - Pricing phase (corresponding to the delay between the publication of the TC opinion and the publication of the reimbursement decision).	Orphan indications reimbursed in France through the standard reimbursement procedure on 1 April 2025
**Criterion n°3**	**National public price** **at the time of drug registration on a standard reimbursement list**	Drug prices are presented in euros excluding taxes. These are national prices applied on the day of each drug first registration on a standard reimbursement list.	

* The list “en sus*” enable hospitals to get a specific reimbursement of a drug in addition to hospitalization tariff. Since 1 January 2022, registration on the list “*en sus*” is based on four criteria defined by decree:

- Medicine mainly used in hospital;

- Major or Important clinical benefit (*SMR majeur ou important*);

- Major to minor clinical added value level (*ASMR I to IV*) or No clinical added value (*ASMR V*) if the relevant comparator are already registered on the list;

- Drug cost exceeds 30% of hospitalization tariff (and cannot be covered by this tariff)

Data extraction was performed from 1 April 2025 to 15 April 2025 using several public domain websites. Public sources were chosen because they provide transparent, reliable and easily accessible information regarding market access for drugs.

Information on access to the French market were obtained from: the French Health Authority website [18], the French national agency for medicines and health product safety website [29], the French public drug database [30] and the Official Journal of the French Republic [31].

For the other European markets, except for Italy, information on drug reimbursement and national public prices in each country, were found on HTA agency websites such as the Gemeinsamer Bundesausschuss website (for Germany) [32], the Ministerio De Sanidad website (for Spain) [33] and the National Institute for Health and Care Excellence (NICE) website (for United Kingdom) [34]. Regarding Italy, these informations were collected from the Gazzetta Ufficiale [35]. In addition, we asked EVERSANA, the owner of NAVLIN, a private database on international drug market access, to provide us with some data to complete our secondary analysis.

Please note that the price comparison analysis was conducted using only one pharmaceutical presentation of each orphan drug for France and the other European countries. For drugs with multiple presentations (e.g. different doses, formulations), the most economic presentation was systematically chosen for the analysis.

## Results

**Fig. 2:**
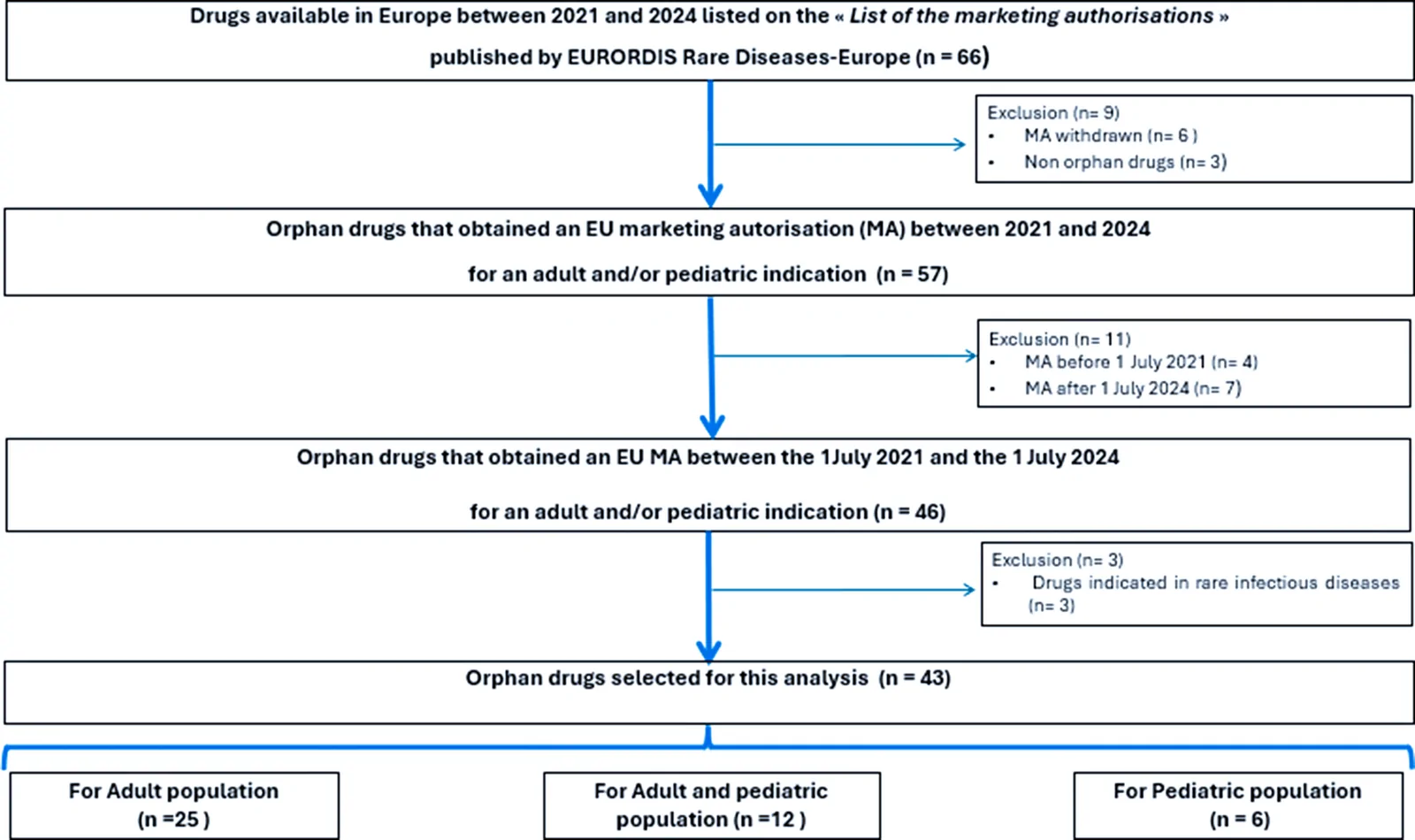
Flow-chart of the orphan drug process selection

Overall, 43 orphan drugs were selected (Fig. 2, Additional file 1) corresponding to 53 marketing authorizations/extension of MA obtained between 1 July 2021 and 1 July 2024. Only 51 MA/extensions of MA were analyzed, as Finlee^®^ (dabrafenib esylate) and Spexotras^®^ (trametinib dimethyl sulfoxide) were considered together when used in a fixed combination for the treatment of high-grade and low-grade glioma.

### French market access

Out of the 51 indications analyzed, only 53% (*n* = 27/51) were reimbursed in France on 1 April 2025 with 57% being pediatric indications (*n* = 4/7) and 48% being adult indications (*n* = 14/29) (Table 2, Additional file 2). Even though the standard reimbursement procedure remains the main way of reimbursement, there is an important number of indications reimbursed through a derogatory access program (44%, *n* = 12/27) with 50% (*n* = 6/12) being reimbursed pre-MA and 42% (*n* = 5/12) post-MA (Table 2). This is because derogatory access programs have been specifically created for drugs indicated for serious and rare diseases. It should be also noted that Voydeya^®^ is the only drug from this sample, with an indication reimbursed through the “direct access” program.

Two reasons may explain why some orphan indications were not reimbursed on 1 April 2025, regardless of the target patient population. First, in most cases, the standard reimbursement procedure is still ongoing after a favorable opinion from the TC (58%, *n* = 14/24) while only a few indications were not recommended for standard reimbursement (12%, *n* = 3/24). Moreover, for several indications, the TC opinion (for standard or derogatory access reimbursement) was not available on the HAS website at the time of analysis (25%, *n* = 6/24). Second, in the case of an ongoing standard reimbursement procedure, these indications were not reimbursed in the meantime through any derogatory access program (either because it was not requested, denied or later withdrawn by HAS) (Table 2).

**Table 2:**
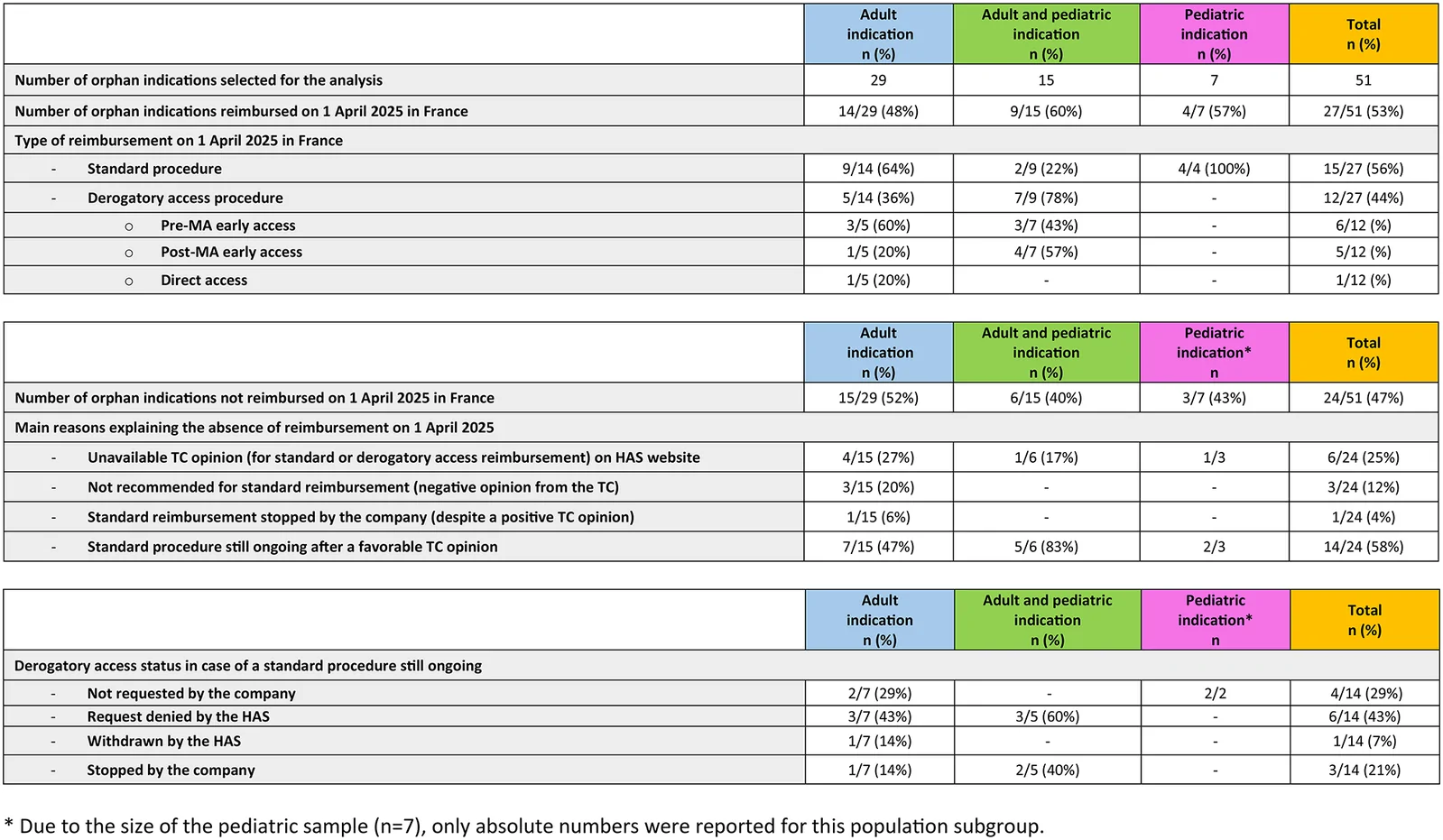
Main results regarding reimbursement in France on 1 April 2025

Among the 15 orphan indications reimbursed through the standard procedure, it is worth noting that Abecma^®^’s pricing phase was very lengthy (1064 days) due to a clock-stop of the procedure. Indeed, initially the TC concluded on an ASMR V for Abecma^®^ which prevented the product to be eligible for the list “*en sus*”. However, Abecma^®^ was already available for patients through derogatory access. As a result, the company used this period to collect new clinical data on Abecma^®^. Based on this data, the TC in a second opinion concluded on an ASMR IV for Abecma^®^. Then, the pricing negotiation restarted and lead to a standard reimbursement with a registration of the product on the list “*en sus*” (after 1083 days of procedure). Thus, due to its exceptional lengthy duration to access reimbursement in France, Abecma^®^ was excluded post-hoc for the different median delay’s calculation.

**Fig. 3:**
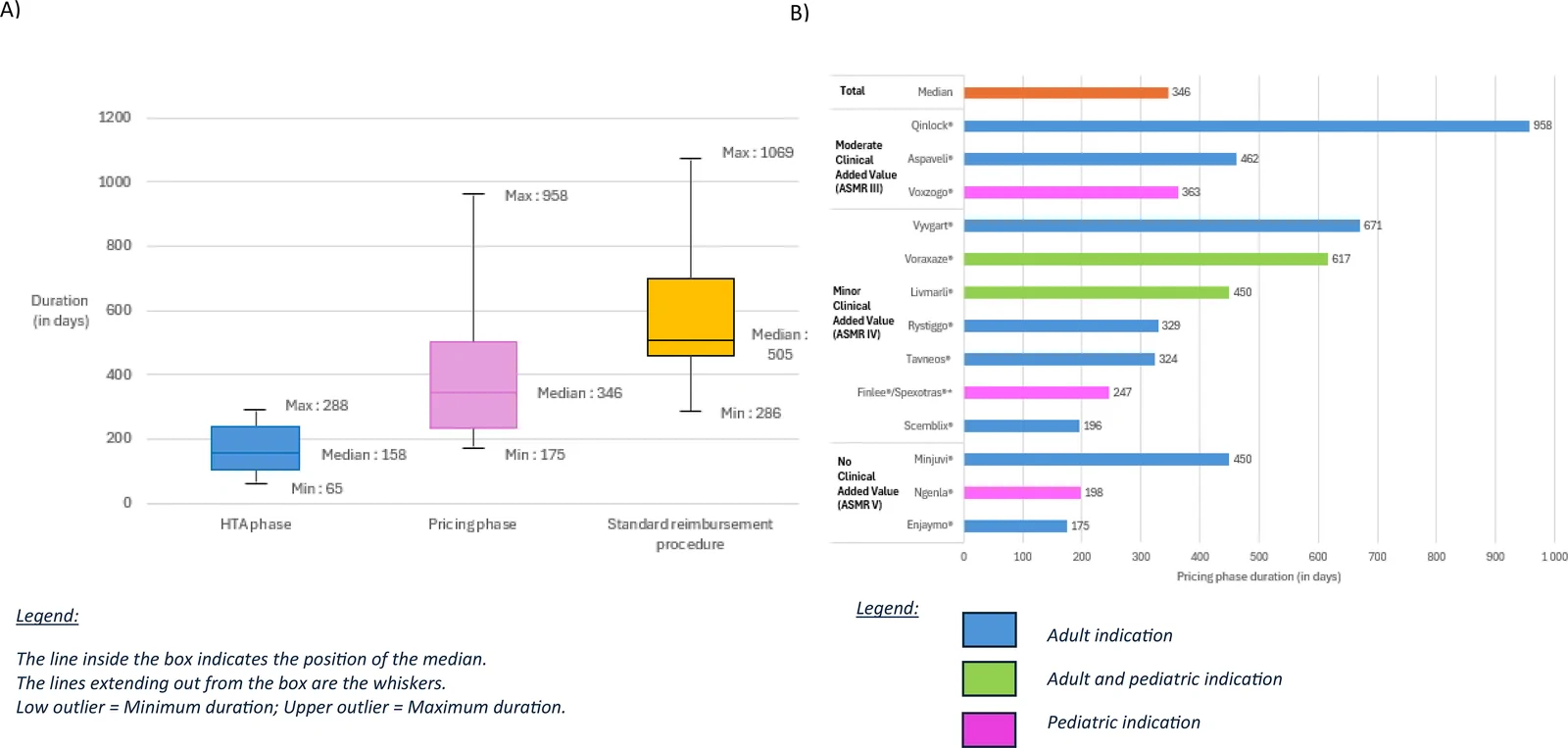
Main results regarding reimbursement delays of drugs reimbursed through the standard procedure on 1 April 2025 (*n* = 14 drugs, after Abecma^®^ exclusion). (**A**) Standard reimbursement procedure, HTA and pricing phase duration. (**B**) Pricing phase duration depending on clinical added value level of the drugs reimbursed

Our post-hoc analysis shows a median time to standard reimbursement of 505 days for these indications (Fig. 3). This delay is largely driven by the pricing process, which has a median duration (of 346 days) three times longer than the duration of the health technology assessment (Fig. 3). Furthermore, except for Abecma^®^, the pricing phase tends to be longer for products with a moderate or minor clinical added value (ASMR III or IV).

Regarding indications first reimbursed through a derogatory access program, the median delay for approval through the standard procedure was 582 days. That was mainly due to the pricing negotiation, which had a median duration of 450 days in these cases (see Additional file 3).

The national public prices at the time of drug registration on a standard reimbursement list in France for the 15 orphan drugs concerned by this procedure vary from €440.71 (minimum) to €240,000.00 (maximum), with a median price of €7,123.29.

### Access comparisons between France and the four other largest European pharmaceutical markets

For these 15 orphan drugs, national public prices at the time of drug registration on a standard reimbursement list across the four other largest pharmaceutical markets fall within roughly the same price range as in France, between €449.92 (minimum) and €350,000.00 (maximum) (Table 3). However, except for three products, France had the lowest national public prices compared to these markets.

**Table 3:**
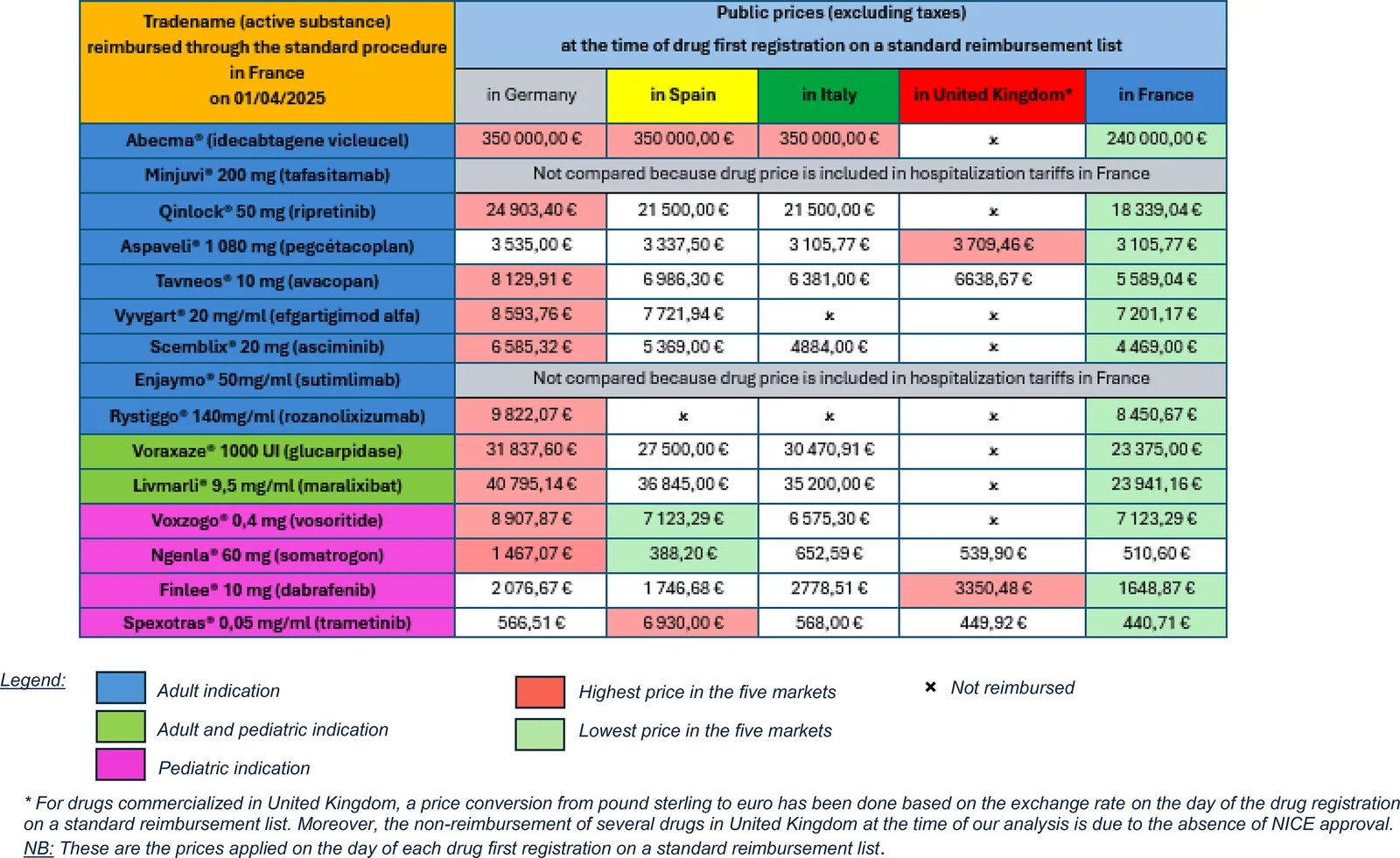
National public prices (excluding taxes) at the time of drug registration on a standard reimbursement list in the five largest European pharmaceutical markets

**Fig. 4:**
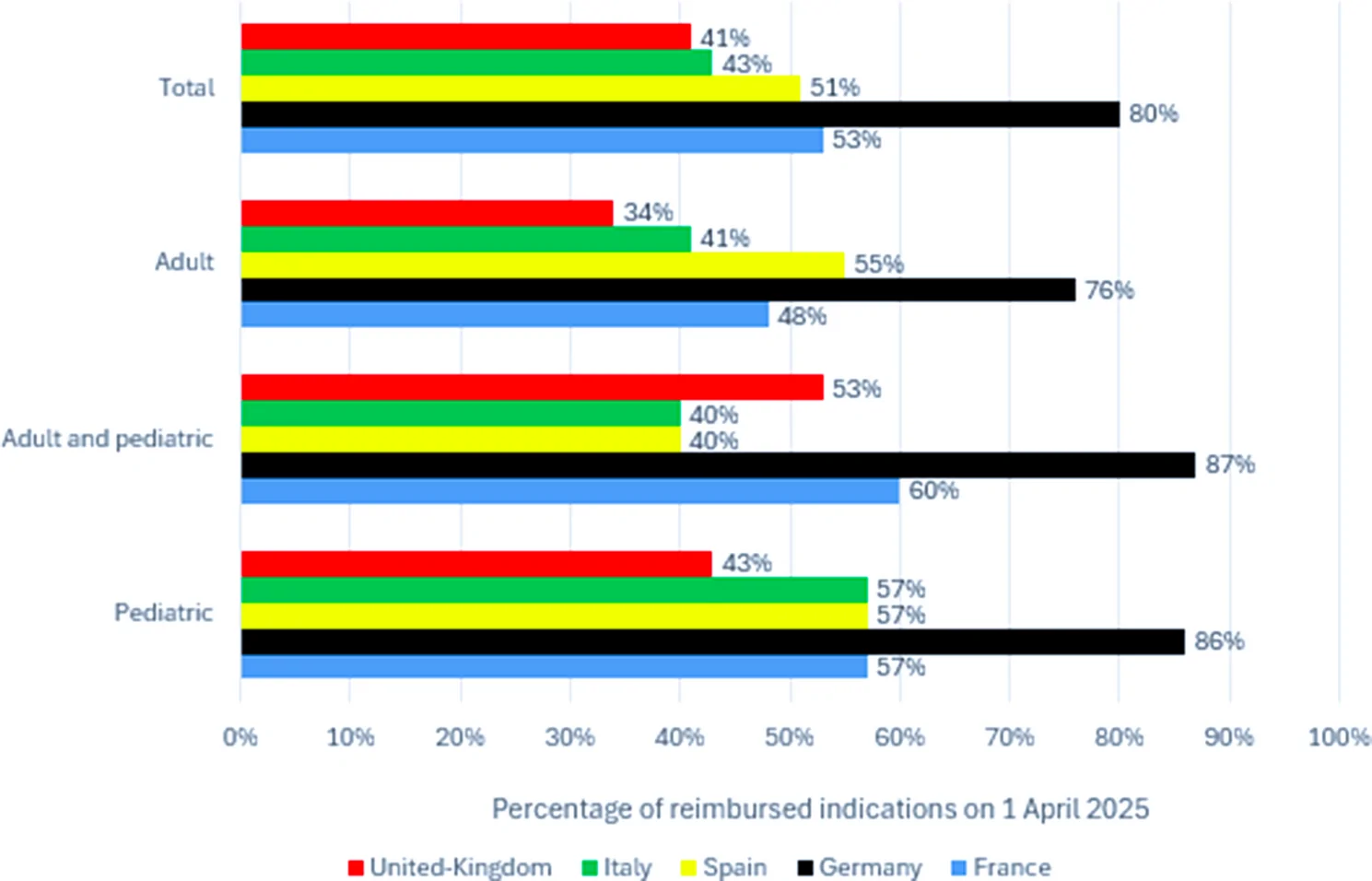
Percentage of reimbursed indications on 1 April 2025 depending on the target population in the five largest European pharmaceutical markets

On 1 April 2025, among the 51 orphan indications included in the analysis, Germany had the highest proportion of reimbursed indications (80%, *n* = 41/51), followed far behind by France (53%, *n* = 27/51), Spain (51%, *n* = 26/51), Italy (43%, *n* = 22/51) and the United Kingdom (41%, *n* = 21/51) (Fig. 4, Additional file 4). This important gap between France and Germany is primarily explained by the fact that market access procedures remain ongoing for many indications in France, whereas in Germany reimbursement is available shortly after the granting of a European marketing authorization.

## Discussion

To our knowledge, this is one of the first studies on French market access for orphan drugs stratifying results regarding the target population of the drug (adults, children or both).

Our study shows that overall, only a part of the selected indications for this analysis were reimbursed (53%, *n* = 27/51) in France on 1 April 2025, with 57% being pediatric indications (4/7) and 48% being adult indications (14/29). However, these results need to be nuanced due to the small number of pediatric indications selected. In this sample there are four times fewer orphan drugs indicated for children than for adults. Moreover, in the study done in 2019 [22], 78.3% of indications were reimbursed in France. The chosen analysis period of market access is one of the reasons that can explain the important difference of reimbursement seen between the two studies. For our study, the analysis period of market access was only 3 years (from 1 July 2021 to 1 July 2024) while in 2019 [22], it was 16 years (because this study focused on all orphan drugs approved before August 2016). Due to maturation time effect, no orphan drug reimbursement trend conclusion in France can be drawn from the comparison alone of these two figures. In addition, please note that reasons explaining the non-reimbursement of indications vary between the two studies. In 2019, it was because the HTA was not finished [22]. For our study, it is rather due to ongoing pricing negotiation and the absence of reimbursement in derogatory access in the meantime (probably because of tighter eligibility criteria for derogatory access since July 2021).

Furthermore, this study shows, regardless of the target population, a median delay to reimbursement through the standard procedure of 505 days, which largely exceeds the European regulatory period of 180 days. This is supported by findings from the 2019 study (the median delay was 360 days) [22]. It should be also noted that, since this study, access delays to drug reimbursement in France tend to be longer. Long delays to reimbursement through the standard procedure are not specific to orphan indications. There are also observed for non-orphan and not related rare diseases indications. Regarding the standard reimbursement procedure for new medicines, this delay has increased from 280 days in 2015 [36] to an average of 508 days in 2021 [37]. Moreover, this study shows a median delay of 346 days for the pricing phase which is particularly long. This longer pricing phase duration trend has been also noticed by the professional organization of pharmaceutical companies operating in France, LEEM (Les entreprises du medicament), in its observatory of 2023, with a median delay of 252 days observed for pricing negotiations [37]. Furthermore, in case of a moderate or a minor clinical added value obtained (*ASMR III or IV*), our study shows a longer pricing phase for these drugs. It may reflect delays in responses from pharmaceutical companies during negotiation rather than difficult discussions due to the drug type (orphan or non-orphan). In its 2023 activity report, CEPS noted that for more than a quarter of drugs valued with an *ASMR III or IV*, the committee had to wait an average of two to nine months, before receiving a counterproposal by pharmaceutical companies [4].

In the case of derogatory access before the reimbursement through the standard procedure, this study shows a median delay between the two types of reimbursement of 582 days mainly due to the pricing phase. This result suggests that derogatory access helps medicines be placed on the market but does not accelerate standard reimbursement procedure, especially drug pricing negotiations. It runs in parallel but does not shorten the pricing phase. In the Income and Expenditure report published by the national health insurance, a median pricing phase of 373 days was observed in the case of derogatory access before the reimbursement through the standard procedure [38].

In addition, our analysis of national public prices at the time of drug registration on a standard reimbursement list for fifteen orphan drugs of this sample, shows very high prices as expected for orphan drugs (Table 3). These public prices are hugely influenced by the reimbursement prices in the other four largest European pharmaceutical markets. Indeed, in several European countries, for some eligible innovative drugs, there is a difference between the public price displayed on Official Journal and the real price paid by the national health insurance. This difference is confidential. In this case, pharmaceutical companies market their medicines at the public price but pay discounts to national health insurance to reach the real price of the drug negotiated.

Intricately linked to reimbursement delays, national public prices of these fifteen drugs in France are lower compared to the other four largest European pharmaceutical markets. Due to the worsening of national health insurance deficit since the COVID-19 crisis, negotiations over reimbursement prices for new drugs have become more difficult and, as a result, longer. These findings are aligned with the results of the 2023–2024 economic balance of the LEEM, which showed 15% to 30% lower prices of essential drugs in France compared to these European markets [39].

Access to treatments for rare diseases in France would be enhanced with shorter times to reimbursement. We suggest three policy measures aimed at improving transparency and reducing negotiation timelines. These reasonable and concrete suggestions could substantially enhance access to drugs. The first solution would be to follow the Spanish example, whereby the agenda of each drug pricing committee meeting is published on the Ministry of Health website. The second solution would be to establish clear deadlines for pricing negotiations at the outset, especially for orphan drugs.

A last solution could be to set a deadline to publish the drug reimbursement decision in the Official Journal and it could be 30 days after a price agreement between the CEPS and the pharmaceutical company. It is worth noting that this last proposal is currently under discussion as part of the renewal of the drug pricing negotiation framework agreement in France.

This study has also certain limitations. Following the approach of the study performed in 2019, only orphan indications were selected for this analysis. So far, most indications in rare diseases treatment do not have an orphan designation in Europe [1]. Moreover, the analysis is cross-sectional at a fixed date (1 April 2025), which means drugs approved near the end of the study period have had less time to complete the reimbursement process, creating a systematic bias toward lower reimbursement rates for more recent drugs. That is why there is probably an underestimation of orphan drug reimbursement in France. Furthermore, it is difficult to determine the real duration of the drug pricing negotiation. For this study, we considered that this phase started from the publication of the HTA of the drug and finished when the Minister of Health decision was published in the official journal. In practice, it may take some time between the end of a pricing negotiation and the publication of the reimbursement decision in the Official Journal, mainly due to administrative constraint (like for example, the signature of reimbursement and pricing decision decrees by the Minister of Health). The long delays observed for this phase are potentially overestimated due to this approximation. Lastly, national public prices were used as an access criterion for this analysis, rather than actual negotiated prices, which means that price comparisons likely overestimate the effective cost difference between countries. Moreover, international comparisons of drug prices are strongly dependent on the timing of the analysis, given the dynamic nature of pharmaceutical pricing. In the case of Abecma^®^, the French price remained aligned with those in other European countries during the period of derogatory access, including after marketing authorization had been granted, but subsequently decreased following its transition to standard reimbursement. The presentation of annual treatment cost would have allowed a better viewing of orphan drug reimbursement impact on national health insurance expenditure and thus on patient’s access.

Future studies on the French market access of drugs in rare diseases, stratifying results regarding drug target population, should include orphan and non-orphan drug to have a representative pediatric sample to study.

## Conclusion

In conclusion, on 1 April 2025 access in France to orphan drugs approved between the 1 July 2021 and the 1 July 2024 was partial regardless of the target population (adults, children or both). Prices and delays associated with the standard reimbursement procedure are the factors that hugely impact the French market access to drugs for rare diseases.

## Supplementary Information

Below is the link to the electronic supplementary material.

Supplementary Material 1

Supplementary Material 2

Supplementary Material 3

Supplementary Material 4

## Data Availability

All data generated or analyzed during this study are included in this published article (and its supplementary information files).

## Footnotes

1 Levels of ASMR possible: major (ASMR I), important (ASMR II), moderate (ASMR III), minor (ASMR IV) and no clinical added value (ASMR V)

## Abbreviations

ASMR: Amélioration du service medical rendu (clinical added value)
CEPS: Comité économique des produits de santé (Economic committee for Health Products)
HAS: Haute autorité de santé (French Health Authority)
LEEM: Les entreprises du médicament (the professional organization of pharmaceutical companies operating in France)
MA: Marketing authorization
SMR: Service médical rendu (clinical benefit)
TC: Commission de la transparence (transparency commitee)
