# Supplementary material for: Reimbursement status, access delays and prices of orphan drugs in France: a population-stratified analysis of indications approved between 2021 and 2024
Source: Orphanet J Rare Dis. 2026 Aug 5;21:393. doi: 10.1186/s13023-026-04533-w (PMC13732822; doi:10.1186/s13023-026-04533-w)
Supplement: Supplementary file 1 [file 13023_2026_4533_MOESM1_ESM.pdf]

Additional Data material 1:

	For Adult population	For Adult and Pediatric population	For Pediatric population
**Pharmaceutical specialty (INN) selected for this analysis**	**Abecma®** (idecabtagene vicleucel) **<br>**Aspaveli®** (pegcetacoplan)**<br>**Carvykti®** (ciltacabtagene autoleucel) **<br>**Enjaymo®** (sutimlimab)<br>**Fabhalta®** (iptacopan)<br>**Filspari®** (sparsentan)<br>**Hemgenix®** (etranacogene dezaparvovec)<br>**Kimmtrak®** (tebentafusp)<br>**Kinpeygo®** (budesonide)<br>**Lunsumio®** (mosunetuzumab)<br>**Minjuvi®** (tafasitamab)<br>**Omjjara®** (momelotinib)<br>**Pyrukynd®** (mitapivat)<br>**Qalsody®** (tofersen)<br>**Qinlock®** (ripretinib)<br>**Rezzayo®** (rezafungin)<br>**Rystiggo®** (rozanolixizumab)<br>**Scemblix®** (asciminib)<br>**Talvey®** (talquetamab)<br>**Tavneos®** (avacopan)<br>**Tepkinly®** (epcoritamab)**<br>**Voydeya®** (danicopan)<br>**Vyvgart®** (efgartigimod alfa)<br>**Yorvipath®** (palopegteriparatide)<br>**Zynyz®** (retifanlimab)	**Agamree®** (vamorolone)<br>**Altuvoct®** (efanesoctocog alfa)<br>**Casgevy®** (exagamglogene autotemcel) **<br>**Filsuvez®** (extrait sec raffiné d'écorce de bouleau)<br>**Hyftor®** (sirolimus)<br>**Imcivree®** (setmelanotide)**<br>**Livmarli®** (maralixibat chloride) **<br>**Loargys®** (pegzilarginase)<br>**Skyclarys®** (omaveloxolone)<br>**Upstaza®** (eladocagene exuparvovec)<br>**Voraxaze®** (glucarpidase)<br>**Zokinvy®** (lonafarnib)	**Finlee®** (dabrafenib esylate) **<br>**Ngenla®** (somatrogon)<br>**Skytrofa®** (lonapegsomatropin)<br>**Spexotras®** (trametinib dimethyl sulfoxidetinib) **<br>**Voxzogo®** (vosoritide)**<br>**Ztalmy®**(ganaxolone)
**Total (%)**	**25/43 (58%)**	**12/43 (28%)**	**6/43 (14%)**

Legend: *** Orphan drug that obtained MA in two therapeutic indications between the 1 July 2021 and the 1July 2024*
