# Supplementary material for: Reimbursement status, access delays and prices of orphan drugs in France: a population-stratified analysis of indications approved between 2021 and 2024
Source: Orphanet J Rare Dis. 2026 Aug 5;21:393. doi: 10.1186/s13023-026-04533-w (PMC13732822; doi:10.1186/s13023-026-04533-w)
Supplement: Supplementary file 2 [file 13023_2026_4533_MOESM2_ESM.pdf]

Additional Data material 2:

A)

	Reimbursement status in France on 1 April 2025		
	Indication Reimbursed		Indication<br>Not Reimbursed
	Through the standard procedure	in Derogatory access	
Pharmaceutical specialty<br>(MA date<br>of the adult indication concerned)	**Abecma**® (18-aug-21)<br>**Minjuvi**® (26-aug-21)<br>**Qinlock**® (18-nov-21)<br>**Aspaveli**® (13-dec-21)<br>**Tavneos**® (11-jan-22)<br>**Vyvgart**® (10-aug-22)<br>**Scemblix**® (25-aug-22)<br>**Enjaymo**® (15-nov-22)<br>**Rystiggo**® (05-janv-24)	**Kimmtrak**® (01-apr-22)<br>**Tepkinly**® (22-sept-23)<br>**Yorvipath**® (17-nov-23)<br>**Voydeya**® (19-apr-24)<br>**Fabhalta**® (17-may-24)	**Carvykti**® (25-may-22 et 19-apr-24)<br>**Lunsumio**® (3-jun-22)<br>**Kinpeygo**® (15-jul-22)<br>**Pyrukynd**® (09-nov-22)<br>**Hemgenix**® (20-feb-23)<br>**Talvey**® (21-aug-23)<br>**Rezzayo**® (22-dec-23)<br>**Omjjara**® (25-jan-24)<br>**Abecma**® (19-mar-24)<br>**Zynyz**® (19-apr-24)<br>**Filspari**® (19-apr-24)<br>**Aspaveli**® (06-may-24)<br>**Qalsody**® (29-may-24)<br>**Tepkinly**® (16-aug-24)
Number of indications/Total number of adult indications selected for this analysis (%)	14/29 (48%)		15/29 (52%)

B)

	Reimbursement status in France on 1 April 2025		
	Indication Reimbursed		Indication Not Reimbursed
	Through the standard procedure	in Derogatory access	
**Pharmaceutical specialty (MA date of the adult and pediatric indication concerned)**	**Voraxaze**® (11-jan-22)<br>**Livmarli**® (09-dec-22)	**Imcivree**® (16-jul-21 et 02-sep-22)<br>**Zokinvy**® (18-jul-22)<br>**Upstaza**® (07-dec-22)<br>**Loargys**® (15-dec-23)<br>**Skyclarys**® (09-feb-24)<br>**Livmarli**® (28-jun-24)	**Filsuvez**® (21-jun-22)<br>**Hyftor**® (15-may-23)<br>**Agamree**® (14-dec-23)<br>**Casgevy**® (09-feb-24 et 09-feb-24)<br>**Altuvoct**® (17-jun-24)
**Number of indications/Total number of adult and pediatric indications selected for this analysis (%)**	9/15 (60%)		6/15 (40%)

C)

	Reimbursement status in France on 1 April 2025		
	Indication Reimbursed		Indication Not Reimbursed
	Through the standard procedure	in Derogatory access	
**Pharmaceutical specialty (MA date of the pediatric indication concerned)**	**Voxzogo**® (26-aug-21)<br>**Ngenla**® (14-feb-22)<br>**Finlee**®**/ Spexotras**® (15-nov-23 /5-jan-24 et 15-nov-23 /5-jan-24)	/	**Skytrofa**® (11-jan-22)<br>**Ztalmy**® (26-jul-23)<br>**Voxzogo**® (24-oct-23)
**Number of indications/Total number of pediatric indications selected for this analysis (%)**	4/7 (57%)		3/7 (%)
