# Supplementary material for: Reimbursement status, access delays and prices of orphan drugs in France: a population-stratified analysis of indications approved between 2021 and 2024
Source: Orphanet J Rare Dis. 2026 Aug 5;21:393. doi: 10.1186/s13023-026-04533-w (PMC13732822; doi:10.1186/s13023-026-04533-w)
Supplement: Supplementary file 3 [file 13023_2026_4533_MOESM3_ESM.pdf]

Additional Data material 3:

Pharmaceutical speciality	Reimbursement decision date in one of the derogatory access pathways created in 2021*	Date of reimbursement through the standard procedure
Abecma®	02-dec-21	13-nov-24
Minjuvi®	27-jan-22	23-jun-23
Qinlock®	18-feb-22	22-oct-24
Aspaveli®	16-feb-22	24-may-23
Tavneos®	17-feb-22	11-aug-23
Vyvgart®	16-dec-21	15-oct-24
Scemblix®	14-apr-22	7-jun-23
Voraxaze®	25-apr-22	28-mar-24
Livmarli®	23-feb-23	17-may-24
Voxzogo®	8-dec-21	13-dec-22
Finlee®/ Spexotras®	29-jun-23	14-mar-25
	29-jun-23	14-mar-25

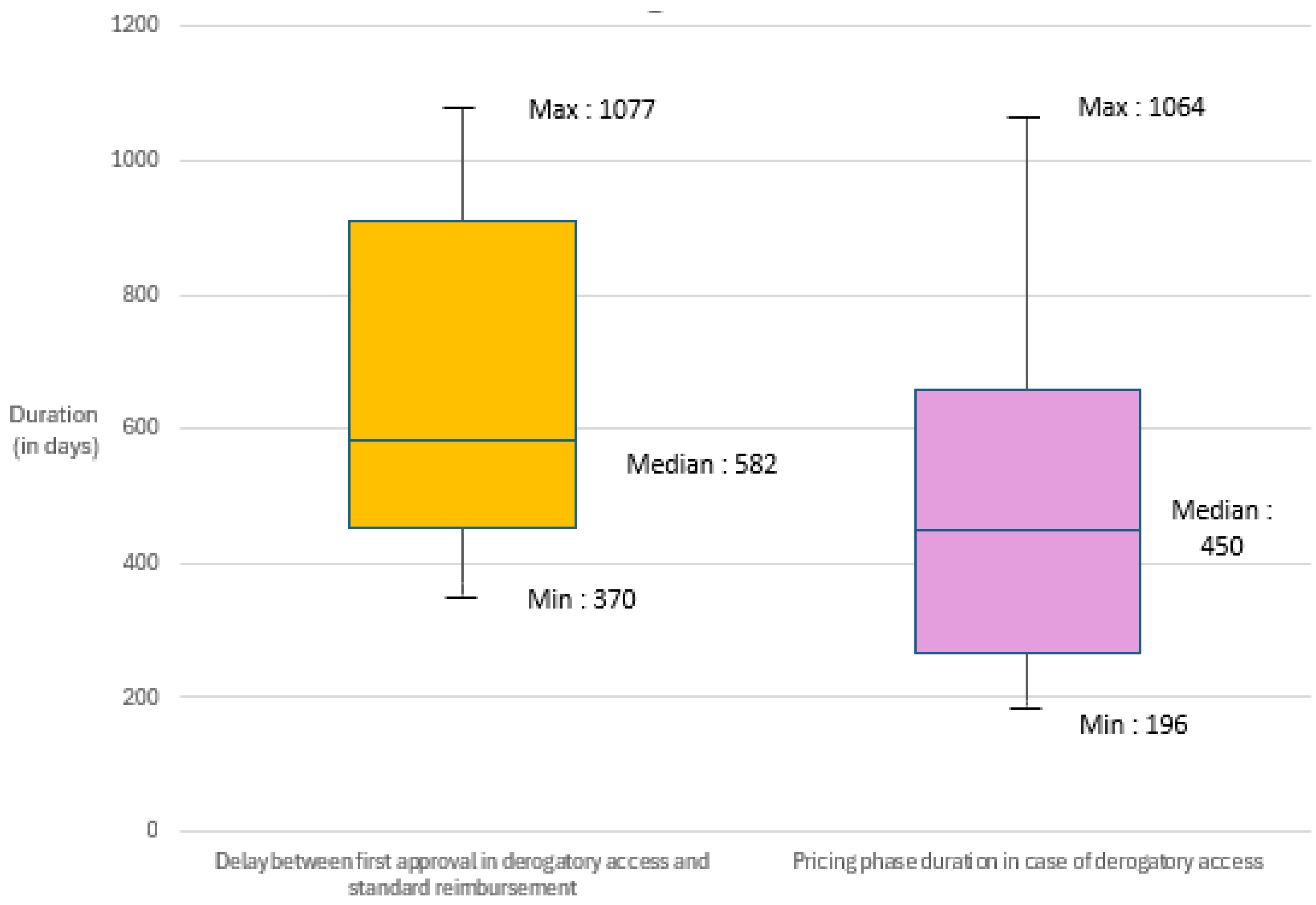

Legend:

- Adult indication
- Adult and pediatric indication
- Pediatric indication

* *Except Abecma®, all these orphan indications were already reimbursed through a temporary authorization of use (ATU) before the reform of the French derogatory access system in July 2021.*
