# Supplementary material for: Reimbursement status, access delays and prices of orphan drugs in France: a population-stratified analysis of indications approved between 2021 and 2024
Source: Orphanet J Rare Dis. 2026 Aug 5;21:393. doi: 10.1186/s13023-026-04533-w (PMC13732822; doi:10.1186/s13023-026-04533-w)
Supplement: Supplementary file 4 [file 13023_2026_4533_MOESM4_ESM.pdf]

Additional Data material 4:

A)

	Germany	Spain	Italy	United-Kingdom	France
Total number of orphan indications reimbursed on 1 April 2025/ Number of orphan indications selected for this analysis (%)	41/51 (80%)	26/51 (51%)	22/51 (43%)	21/51 (41%)	27/51 (53%)

B)

Pharmaceutical Specialty	MA date of the indication	Reimbursement status on 1 April 2025 depending on the country				
		Germany	Spain	Italy	United-Kingdom	France
Abecma®	18-aug-21	✗	✓	✓	✗	✓
	19-mar-24	✓	✗	✗	✗	✗
Minjuvi®	26-aug-21	✓	✓	✓	✗	✓
Qinlock®	18-nov-21	✓	✓	✓	✗	✓
Aspaveli®	13-dec-21	✓	✓	✓	✓	✓
	06-may-24	✓	✗	✗	✗	✗
Tavneos®	11-jan-22	✓	✓	✓	✓	✓
Kimmtrak®	01-apr-22	✓	✓	✓	✓	✓
Carvykti®	25-may-22	✓	✗	✗	✗	✗
	19-apr-24	✗	✓	✗	✗	✗
Lunsumio®	03-jun-22	✓	✓	✓	✗	✗
Kinpeygo®	15-jul-22	✗	✗	✗	✓	✗
Vyvgart®	10-aug-22	✓	✓	✓	✗	✓
Scemblix®	25-aug-22	✓	✓	✓	✓	✓
Pyrukynd®	09-nov-22	✗	✗	✗	✗	✗
Enjaymo®	15-nov-22	✓	✗	✓	✗	✓
Hemgenix®	20-feb-23	✗	✓	✗	✓	✗
Talvey®	21-aug-23	✓	✓	✗	✗	✗
Tepkinly®	22-sep-23	✓	✓	✓	✓	✓
	16-aug-24	✗	✗	✗	✗	✗
Yorvipath®	17-nov-23	✓	✗	✗	✗	✓
Rezzayo®	22-dec-23	✓	✓	✗	✗	✗
Rystiggo®	05-jan-24	✓	✗	✗	✗	✓
Omjjara®	25-jan-24	✓	✓	✓	✓	✗
Zynyz®	19-apr-24	✗	✗	✗	✗	✗
Voydeya®	19-apr-24	✓	✗	✗	✓	✓
Filspari®	19-apr-24	✓	✗	✗	✗	✗
Fabhalta®	17-may-24	✓	✗	✗	✓	✓
Qalsody®	29-may-24	✓	✗	✗	✗	✗
Number of reimbursed indications/ Total number of adult indications selected for this analysis (%)		22/29 (76%)	16/29 (55%)	12/29 (41%)	10/29 (34%)	14/29 (48%)

C)

Pharmaceutical Specialty	MA date of the indication	Reimbursement status on 1 April 2025 Depending on the country				
		Germany	Spain	Italy	United-Kingdom	France
Imcivree®	16-jul-21	✓	✓	✓	✓	✓
	02-sep-22	✓	✗	✗	✓	✓
Voraxaze®	11-jan-22	✓	✓	✓	✗	✓
Filsuvez®	21-jun-22	✓	✓	✗	✓	✗
Zokinvy®	18-jul-22	✓	✗	✗	✗	✓
Upstaza®	07-dec-22	✓	✗	✓	✓	✓
Livmarli®	09-dec-22	✓	✓	✓	✗	✓
	28-jun-24	✓	✗	✗	✗	✓
Hyftor®	15-may-23	✓	✗	✗	✗	✗
Agamree®	14-dec-23	✓	✗	✗	✓	✗
Loargys®	15-dec-23	✓	✓	✓	✗	✓
Skyclaris®	09-feb-24	✓	✗	✓	✗	✓
Casgevy®	09-feb-24	✗	✗	✗	✓	✗
	09-feb-24	✗	✗	✗	✓	✗
Altuvoct®	17-jun-24	✓	✓	✗	✓	✗
Number of reimbursed indications / Total number of adult and pediatric indications selected for this analysis (%)		13/15 (87%)	6/15 (40%)	6/15 (40%)	8/15 (53%)	9/15 (60%)

D)

Pharmaceutical Specialty	MA date of the indication	Reimbursement status on 1 April 2025 depending on the country				
		Germany	Spain	Italy	United-Kingdom	France
Voxzogo®	26-aug-21	✓	✓	✓	✗	✓
	24-oct-23	✓	✗	✗	✗	✗
Skytrofa®	11-jan-22	✓	✗	✗	✗	✗
Ngenla®	14-feb-22	✓	✓	✓	✓	✓
Ztalmy®	26-jul-23	✗	✗	✗	✗	✗
Finlee®/ Spexotras®	15-nov-23/ 5-jan-24	✓	✓	✓	✓	✓
	15-nov-23/ 5-jan-24	✓	✓	✓	✓	✓
Number of reimbursed indications / Total number of pediatric indications selected for this analysis (%)		6/7 (86%)	4/7 (57%)	4/7 (57%)	3/7 (43%)	4/7 (57%)

Legend:

- (blue) *Adult indication*
- (green) *Adult and pediatric indication*
- (pink) *Pediatric indication*
- ✓ *Reimbursed*
- ✗ *Not reimbursed*
